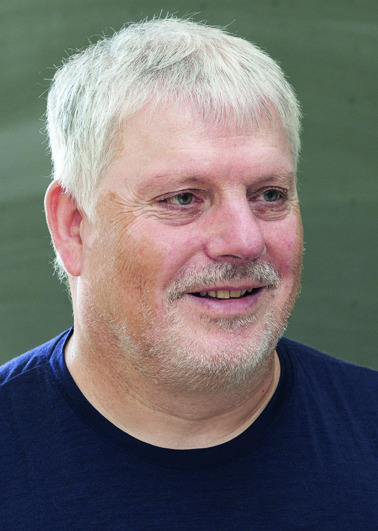# Electrocatalytic Ammonia Oxidation Reaction: Selective Formation of Nitrite and Nitrate as Value‐Added Products

**DOI:** 10.1002/cssc.202402516

**Published:** 2025-03-28

**Authors:** Ieva A. Cechanaviciute, Wolfgang Schuhmann

**Affiliations:** ^1^ Analytical Chemistry – Center for Electrochemical Sciences (CES) Faculty of Chemistry and Biochemistry Ruhr University Bochum Universitätsstr. 150 D-44780 Bochum Germany

**Keywords:** ammonia oxidation reaction, nitrite/nitrate, Ostwald process, hydrogen carrier, electrocatalysis

## Abstract

Ammonia (NH_3_) plays a pivotal role as a hydrogen carrier, offering a carbon‐free energy alternative for sustainable energy systems. The ammonia electrooxidation reaction (AmOR) emerges as a promising avenue to leverage NH₃ in energy conversion and environmental applications. This review explores the multifaceted importance of NH_3_ oxidation through three primary strategies: its integration into fuel cell technology for clean energy generation, its use in wastewater treatment for ammonia removal, and its application in electrolyzer setups for producing value‐added products. Special emphasis is placed on oxidizing NH_3_ to nitrite (NO_2_^−^) and nitrate (NO_3_^−^) in electrolyzers as a potential alternative to the energy‐intensive Ostwald process. The review highlights recent advances in catalyst development for efficient NO_2_^−^/NO_3_^−^ synthesis, the influence of the pH on reaction selectivity, and various reported experimental AmOR solutions. By addressing these critical aspects, this work aims to underscore the potential of NH_3_ oxidation in electrolyzers for sustainable energy solutions. Potential future research directions and challenges are also discussed.

## Graphical abstract

Ammonia (NH_3_) is an important hydrogen carrier with applications in energy and chemical production. This review highlights NH_3_ electrooxidation strategies, including fuel cells, wastewater treatment, and electrolyzers for nitrite (NO_2_^−^) and nitrate (NO_3_^−^) production as an alternative to the Ostwald process. Key topics include an overview of non‐noble catalysts, pH effects, and measurement design optimization for sustainable and efficient NH_3_ utilization.

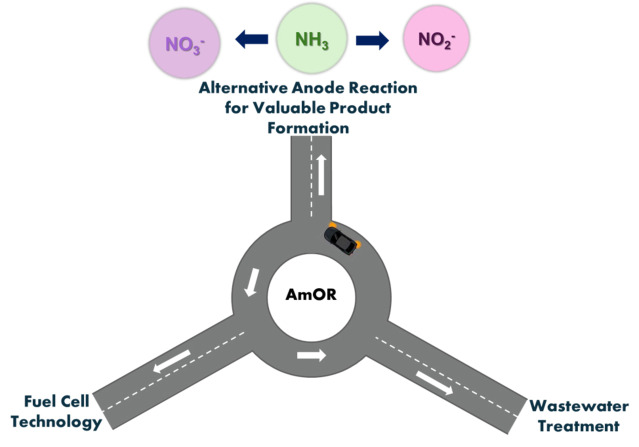

## 1 Introduction

Despite the move for sustainability global energy supply relies heavily on fossil fuels. However, wind and solar‐based renewable energy technologies can at least partially mitigate the dependence on carbon‐based fuels and their negative impact on the environment.^[1]^ Although highly beneficial and widely implemented, solar and wind power is not without drawbacks. Solar and wind‐driven electricity generation depends on the intensity of sunlight and wind, respectively. Therefore, varying weather conditions cause intermittency in power output. Power‐to‐X strategies were proposed as an alternative, where renewable electricity surplus is converted into various energy carriers.^[2]^ Arguably one of the most widely discussed power‐to‐X pathways is the generation of hydrogen (H_2_) during water electrolysis. Hydrogen is an attractive fuel of the future as it can be used for energy storage and no greenhouse emission is produced during hydrogen oxidation in fuel cells.^[3]^

In recent years, multiple projects and research studies proposed that green hydrogen could be produced in remote areas where renewable energy sources are plentiful, e. g. deserts.[^4^, ^5^] This way, not only the surplus, but the majority of solar electricity would be converted to chemical energy. Presently, the majority of hydrogen is obtained through high amounts of carbon oxides (CO_x_) emitting processes, namely steam methane reforming (SMR) and coal gasification, that can be additionally assisted with the water‐gas shift reaction.[^6^, ^7^, ^8^] Therefore, the move for mass production of green hydrogen would accelerate hydrogen application as a fuel for fuel cell technologies, and reduce the extensive application of the aforementioned environmentally demanding technologies. However, a major obstruction on the way to global hydrogen supply and utilization is its transportation.^[9]^ Gaseous hydrogen has a very low energy density per unit volume. Therefore, it needs to be compressed or liquified to enable large‐scale transportation. Both compression (350‐700 bar) and liquefaction (−253 °C) are energy intensive and negatively impact the process efficiency and economic value.[^10^, ^11^] Moreover, hydrogen molecules are small, making them very leak‐prone. The possibility of leaks combined with the explosivity of hydrogen raises safety concerns, that require rigorous measures and monitoring systems to be addressed.[^12^, ^13^] Another drawback that should not be overlooked is the underdeveloped hydrogen transportation infrastructure – whereas natural gas or oil has extensive pipeline networks, hydrogen pipelines are relatively rare.^[14]^

Multiple studies proposed to replace hydrogen by hydrogen carrier compounds to mitigate limitations related to hydrogen transportation.^[15]^ Some of the suggested alternatives include organic materials such as methylcyclohexane, toluene, methanol, or formic acid.[^16^, ^17^] However, these materials do not fulfil the requirements for carbon neutrality, as processing organic compounds would inevitably lead to the formation of carbon oxides. Therefore, 17.7 wt% hydrogen containing ammonia (NH_3_) became widely discussed as the most promising hydrogen carrier/storage compound. It can be liquefied at a significantly lower temperature of only −33 °C at atmospheric pressure and contains higher volumetric energy density than hydrogen.[^18^, ^19^] Moreover, it is non‐flammable, which significantly reduces safety concerns during transport and storage. For decades ammonia has been one of the most mass‐produced chemicals in the world because of its importance in fertilizer production. Correspondingly, global ammonia transportation infrastructure is already well‐established.^[20]^

Ammonia is usually produced in large industrial plants using the Haber‐Bosch (HB) process. Fritz Haber developed a method for ammonia synthesis from hydrogen and nitrogen (N_2_) under high pressure and temperature in 1909.^[21]^ Later, Carl Bosch industrialized this process, with the first synthesis plant being built in 1911. The HB process operates at temperatures in the range of 400–500 °C and pressures in the range of 150–300 bar, usually in the presence of an Fe‐based catalyst.^[22]^ The environmental impact of the HB process could be significantly reduced by utilizing green hydrogen obtained via electrolysis compared to the hydrogen obtained via SMR and coal gasification routes. The process ideally could also be powered using renewable energy.^[23]^ As an alternative to HB, electrocatalytic nitrogen reduction to ammonia is highly investigated.[^24^, ^25^, ^26^] Unfortunately, at the present state, the reported production rates are far too low to have considerable significance on the roadmap of the ammonia economy.

Although green ammonia itself could be treated as a chemical of interest, in the scope of this review, ammonia is viewed as a hydrogen energy storage compound and fuel. In recent years many in‐depth studies have been published highlighting the challenges and opportunities of ammonia‐based energy production, consumption, storage, transportation, and economics.[^18^, ^19^, ^20^, ^27^, ^28^, ^29^, ^30^, ^31^, ^32^, ^33^] Ammonia oxidation, which is closely related to the topic of ammonia energy, received similarly great attention.[^34^, ^35^] Ammonia can be split back into nitrogen and hydrogen through a variety of methods. Upon heating to temperatures of 400 °C and above thermal decomposition of ammonia can occur.^[36]^ However, the requirement for high temperatures can contribute significantly to energy‐efficiency loss. Ammonia combustion engines were also proposed as a way to utilize it as a fuel.^[37]^ However, ammonia has a lower energy content in comparison with fuels such as gasoline or diesel, is difficult to ignite due to its high octane number, and might present a high probability of harmful nitrogen oxide (NO_x_) production. Electrocatalytic ammonia oxidation reaction (AmOR) presents an alternative pathway for ammonia utilization either by employing fuel cell or electrolyzer technologies.[^38^, ^39^] The most expected and commonly desired product during AmOR is nitrogen. However, more recently the advantages of nitrite (NO_2_^−^) and nitrate (NO_3_^−^) production were also acknowledged.^[40]^ In the present review, different perspectives related to AmOR are discussed. However, special focus is placed on the advantages and existing barriers in selective NO_2_^−^/NO_3_^−^ formation as an alternative to the environmentally and energetically demanding Ostwald process. The study highlights the industrial importance of nitrite and nitrate, possible selectivity‐enhancing strategies, and presents an overview of already reported catalyst materials.

## 2 Application‐Dependent Selectivity Requirements

The AmOR is generally investigated in the context of either fuel cell or electrolyzer technologies. Most commonly, for the application in fuel cells, ammonia is considered a fuel that can be oxidized to nitrogen via a 6 e^−^ transfer process, as previously reported by multiple reviews.[^41^, ^42^] As an alternative anode reaction in an electrolyzer setup, AmOR could replace the high potential oxygen evolution reaction (OER), increasing water splitting efficiency. This concept has been applied to ammonia removal from wastewater, where the formation of environmentally neutral nitrogen is also desirable. Multiple reviews discussing the specifics of AmOR in relation to wastewater treatment have been published as well.[^43^, ^44^, ^45^] Wastewater contamination is closely linked to the nitrogen cycle, which is strongly affected by human activity. It could be expected that increasing ammonia formation would further affect the nitrogen cycle, making the control over nitrogen species even more crucial. Following a more novel approach, ammonia in an electrolyzer setup could be converted to value‐added products, namely nitrite and nitrate, simultaneously producing hydrogen at the cathode.^[46]^ The following section seeks to further highlight the different potential applications and advantages provided by each approach. Finally, it is necessary to acknowledge that regardless of the discussed distinct perspectives on AmOR, other interesting strategies might also exist, e. g. ammonia‐contaminated wastewater supply as a fuel for ammonia fuel cells.^[47]^

### 2.1 Ammonia Fuel Cells

Proton‐exchange membrane fuel cells (PEMFC) are not compatible with ammonia due to the acidic nature of the electrolyte and the used cation‐exchanging membrane. Therefore, ammonia first needs to be decomposed to nitrogen and hydrogen, the latter then participating in the hydrogen oxidation reaction (HOR).[^48^, ^49^] As ammonia is not directly consumed as a fuel, these concepts are called indirect ammonia fuel cells. Indirect mechanisms require thermal ammonia splitting and a high‐purity ammonia‐free hydrogen supply, which together limit the efficiency of the process. Direct ammonia fuel cells (DAmFC) provide an alternative operating principle, where NH_3_ can be directly processed in the fuel cell. Alkaline, molten alkaline, and anion‐exchange membrane (AEM) technologies have been suggested as low‐temperature DAmFCs.[^50^, ^51^] All alkaline‐based DAmFCs operate following a similar principle, where OH^−^ ions react with ammonia at the anode and oxygen is reduced to OH^−^ at the cathode. Due to the properties of the AEM membrane, AEM‐DAmFCs cannot be operated at high temperatures (>120 °C) as alkaline and molten alkaline cells; however, they are significantly more tolerant to CO_2_ poisoning, which is a well‐known issue in alkaline fuel cell technology.^[38]^ Solid oxide fuel cells (SOFC) that usually operate at a temperature range from 450 °C to 900 °C are considered high‐temperature DAmFCs.[^52^, ^53^] SOFCs use solid‐state conductive materials where either oxygen anion (SOFC−O) or protons (SOFC−H) are conducted as electron carriers. High temperatures facilitate ammonia cracking and obtain hydrogen then reacts with O^2−^ or oxidizes to H^+^ depending on the type of SOFC. While a SOFC demonstrates high peak power density, a major drawback of SOCFs is long‐term stability and a sluggish start‐stop process, which also limits the possible implementation of the technology in e. g. automotive industry.^[54]^

The formation of oxidized nitrogen compounds (NO_x_) during AmOR might have negative implications on the DAmFC performance. Selective N_2_ formation also ensures environmentally compatible selectivity. One of the most widely investigated catalysts for low‐temperature DAmFCs is Pt, specifically the superior‐performing (100) facets.[^34^, ^55^, ^56^, ^57^, ^58^, ^59^] Although Pt has a high affinity for ammonia and demonstrates good AmOR performance, factors such as high cost and *N adsorption‐related surface poisoning prompt a further search for alternative catalyst materials. Combining Pt with other noble (Ir, Rh, Ru, Pd, Au)[^60^, ^61^, ^62^] and/or non‐noble (Zn, Ce, Ni)[^63^, ^64^, ^65^] metals has proven to be a viable strategy to improve the dehydrogenation of ammonia to NH_x_ species and prevent catalyst poisoning. Noble‐metal‐free catalyst materials often based on Fe, Co, Cu, and Ni were reported for DAmFCs as well.[^66^, ^67^] The Ni‐based catalyst activity was even reported to surpass the one of commercial Pt/C.^[68]^ However, no analysis of reaction products was performed, which makes the application of such materials for low‐temperature DAmFC questionable, as Ni is also known to be a NO_2_^−^/NO_3_^−^‐forming catalyst.^[69]^ Interestingly, multiple studies have also highlighted the positive influence on selectivity and/or activity when combining multiple metals in a catalyst.[^70^, ^71^] Nevertheless, the application of low‐temperature DAmFCs is hindered due to the sluggish kinetics of AmOR in comparison to HOR. Therefore, further investigation of catalyst materials and their activity and selectivity is still crucial to improve the efficiency of DAmFCs.

### 2.2 Wastewater Treatment

Ammonia plays a vital role in the nitrogen cycle, a biochemical process where nitrogen is transformed into various chemical forms as it moves through the atmosphere and different ecosystems. Before the global adoption of the HB process, nearly all reactive nitrogen species were produced and recycled by microorganisms.^[72]^ While the widespread use of synthetic fertilizers is essential for sustaining nearly half of the global population, it has also disrupted the nitrogen cycle.^[73]^ Therefore, electrochemical methods for converting nitrogen‐containing species have been explored as a way to replicate natural microorganism‐driven processes and mitigate the negative effects of human activity.

Although widely applied, ammonia is toxic in water or the bloodstream, and can disrupt cellular functions at elevated levels, leading to harmful effects on aquatic organisms and human health.[^74^, ^75^] Ammonia reaches wastewater mainly through domestic sewage, industrial activities, agricultural runoff, and the decomposition of organic materials. Other common ammonia removal methods include microbial processing, membrane separation, adsorption, ion exchange, chemical precipitation, ammonia stripping, chlorination, or oxidation using other chemical species, and photocatalysis.[^43^, ^76^] Both direct AmOR on the catalyst surface and chlorine‐mediated indirect AmOR have been reported as alternatives to traditional methods. In the case of the indirect reaction pathway, hydroxyl radicals and active chlorine species generated during chlorine evolution initiate the oxidation of ammonia.^[77]^ However, direct electrocatalytic ammonia oxidation provides a fast, efficient, and chemical‐free alternative, where, with correct selectivity control, no harmful by‐products or excess sludge is produced. Its versatility and minimal maintenance needs could make it a superior alternative to traditional methods like biological processing. As already mentioned previously, due to the environmental compatibility the wastewater treatment strategy would mostly benefit from selective ammonia conversion to nitrogen. Although the earliest reports were focused on ammonia oxidation for fuel cells on Pt,[^78^, ^79^, ^80^, ^81^] Marincic *et al*. proposed electrolysis as a method for ammonia wastewater treatment on non‐noble metal electrodes as early as 1978.^[82]^ For a commercial wastewater treatment facility working close‐to‐neutral pH could be advantageous. Therefore, it should be considered that AmOR selectivity and removal efficiency were reported to be pH‐dependent.^[83]^ The pKa (9.25) value of the NH_4_^+^/NH_3_ equilibrium should also be taken into consideration to determine which form of active species is participating in a reaction. However, even in highly alkaline pH (11.8 – 13.5) environments, where only NH_3_ exists, AmOR on Pt (100) was demonstrated to be pH‐dependent, with the detectable formation of NO species at pH >12.^[84]^ Some studies successfully investigated AmOR in a near‐neutral pH environment using real‐life samples e. g. graphite electrodes for urine sample treatment or Ti/PbO_2_ electrodes for ammonia removal from wastewater.[^85^, ^86^] Quan *et al*. even reported a single‐atom Fe‐based catalyst with high ammonia removal efficiency and up to 99 % ammonia conversion selectivity to N_2_ in a pH 7 electrolyte.^[87]^ A more unconventional catalyst composition was reported by Hu *et al*., using a high‐entropy spinel oxide (MnFeCoNiCu)_3_O_4_ that significantly outperformed the activity of its constituent elements and reached a 90 % FE value for N_2_ formation.^[88]^

### 2.3 Alternative Anode Reaction for Valuable Product Formation

The primary goal of water electrolysis is to produce hydrogen through an environmentally sustainable route. Currently, oxygen is produced at the anode as a byproduct, but it holds limited economic value. Additionally, the slow reaction kinetics and high overpotentials associated with the OER reduce the overall energy efficiency of the process. During hybrid water electrolysis, the OER can be substituted with a thermodynamically more favorable reaction, improving energy efficiency and lowering the cost of producing green hydrogen. Ammonia wastewater treatment can also be considered as an alternative anode reaction during hybrid water electrolysis. However, as nitrate groundwater contamination is a serious environmental problem, nitrate production during wastewater treatment would require additional processing.^[89]^ Ammonia, as a valuable chemical, could be recovered and reused, instead of being converted to nitrogen as well.^[90]^ Nevertheless, different strategies are necessary when discussing AmOR in the context of ammonia as a hydrogen carrier. Ammonia produced at an appropriate location through a green Haber–Bosch process could be transported to a modified LNG terminal. There, the ammonia is converted into gas and supplied either to a pipeline or directly to an electrolyzer system. By utilizing additional green electricity, the amount of hydrogen generated would be significantly increased at a lower applied potential compared to OER, with the simultaneous production of nitrite or nitrate, which are valuable industrial compounds that are normally obtained through the Ostwald process. The Ostwald process enables the production of nitric acid (HNO_3_) and related chemicals by oxidizing ammonia with oxygen usually employing a noble metal catalyst (Pt−Rh) at high temperatures. First, ammonia is oxidized to nitrogen monoxide (NO), which is then further oxidized to nitrogen dioxide (NO_2_) that can be absorbed in water, obtaining nitric acid. Compounds like sodium nitrite (NaNO_2_) and sodium nitrate (NaNO_3_) are obtained from Ostwald process‐related reactions. Nitrites and nitrates are essential for manufacturing fertilizers, various chemicals, and food preservatives, making the Ostwald process critical for global agriculture and industry. However, the process releases nitrogen oxides (NO_x_), which are potent greenhouse gases and contribute to air pollution, posing environmental and health hazards. Selective electrocatalytic AmOR could enable the synthesis of nitrite and/or nitrate that is dissolved in the electrolyte avaiding the production of gaseous nitrogen oxides. Additionally, the high temperatures and use of precious metals as catalysts make the process energy‐intensive and costly.[^91^, ^92^, ^93^]

Ammonia conversion to NO_2_^−^ or NO_3_^−^ could partially alleviate the reliance on the Ostwald process, simultaneously assisting hydrogen generation. Still, the strategy described is not without its challenges. Formation of NO_x_ species occurs through complicated reaction pathways which often require high potentials, increasing the competition with the OER. Therefore, the research area of electrocatalysts for selective nitrite/nitrate formation from NH_3_ is rapidly developing.^[40]^

## 3 Proposed AmOR Mechanisms

### 3.1 Nitrogen Formation

Two primary mechanisms for NH_3_ oxidation to N_2_ were suggested: the Oswin‐Salomon^[80]^ and the Gerischer‐Mauerer mechanisms.^[81]^ The Oswin‐Salomon mechanism suggests that the reaction proceeds via a complete, stepwise dehydrogenation at the nitrogen atom, leading to the formation of dinitrogen through the interaction of two adsorbed nitrogen species (*N) (Equations (1.1)–(1.4)). In contrast, Gerischer and Mauerer proposed an alternative route where partially dehydrogenated species significantly contribute to N_2_ formation (Equations (2.1)–(2.3)). Moreover, as suggested by Gerischer and Mauerer the formation of *N species is contributing to catalyst activity loss, due to the poisoning effect.^[94]^ However, some studies also reported that the ability of Pt to overcome poisoning by adsorbed nitrogen species is highly dependent on its crystal structure, this way explaining the superior activity of Pt (100) in comparison to Pt (111).[^58^, ^95^, ^96^] Some studies have also proposed that the passivation effect originates from the oxidation of the metal surface or adsorbed *NO species formation.[^97^, ^98^] 
(1.1)

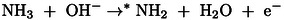



(1.2)

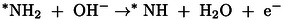



(1.3)

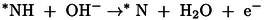



(1.4)

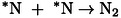



(2.1)

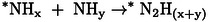



(2.2)

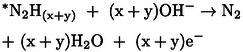



(2.3)

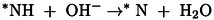




Both mechanisms were first discussed and most extensively investigated on Pt catalysts.^[99]^ The Gerischer‐Mauerer mechanism is more widely accepted with multiple studies indicating that N−N bond formation occurs through the reaction of hydrogenated nitrogen species.[^59^, ^100^, ^101^, ^102^] However, Ishikawa and colleagues found that on Pt(100), AmOR is potential‐dependent, following the Gerischer‐Mauerer pathway at low potentials (<0.5 V vs. RHE) and the Oswin‐Salomon mechanism at higher potentials (≥ 0.5 V vs. RHE).^[103]^ A recent study by Venturini *et al*. investigated the potential‐dependent AmOR mechanism on Pt using online electrochemical mass spectrometry (OLEMS) and ion chromatography (IC).^[104]^ The study demonstrated that oxidized nitrogen species such as NO can also be formed during AmOR in a relatively low potential region of 0.45 V‐0.8 V vs. RHE, which was initially thought to be dominated by N_2_ formation. Moreover, the authors also summarized literature results obtained by other researchers and highlighted that although not without similarities, significant differences in selectivity tendencies can also be observed during operando investigations of AmOR on Pt which may be explained by variations in the Pt structure. This highlights, that although the N_2_ formation mechanism is much better understood compared to NO_x_ pathways, it is not without discussions.

### 3.2 Formation of Oxidized Nitrogen Species

The majority of the available studies discussing AmOR mechanisms are focused on the nitrogen formation pathways. However, more recently some researchers also attempted to deconvolute the pathway of oxidized nitrogen species formation.

Ammonia conversion to NO_2_^−^ or NO_3_^−^ is known to be 6 e^−^ (3.1) or 8 e^−^ (3.2) process, respectively. However, there is no consensus on step‐wise mechanistic reaction pathways. Formed nitrous oxides can react with ammonia, and each other, or the produced oxygen, when venturing into the OER region. This makes deconvoluting the AmOR mechanism an especially challenging task. To the best of our knowledge, available computational and experimental studies confirm that N_2_ is energetically the most favorable product, and the predominant formation of NO_2_^−^ or NO_3_^−^ is only achieved at higher oxidative potentials. Correspondingly, higher oxidative potentials were generally reported for the formation of NO_3_^−^ in comparison to NO_2_^−^.
(3.1)

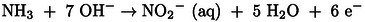



(3.2)

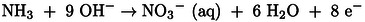




**Figure 1:**
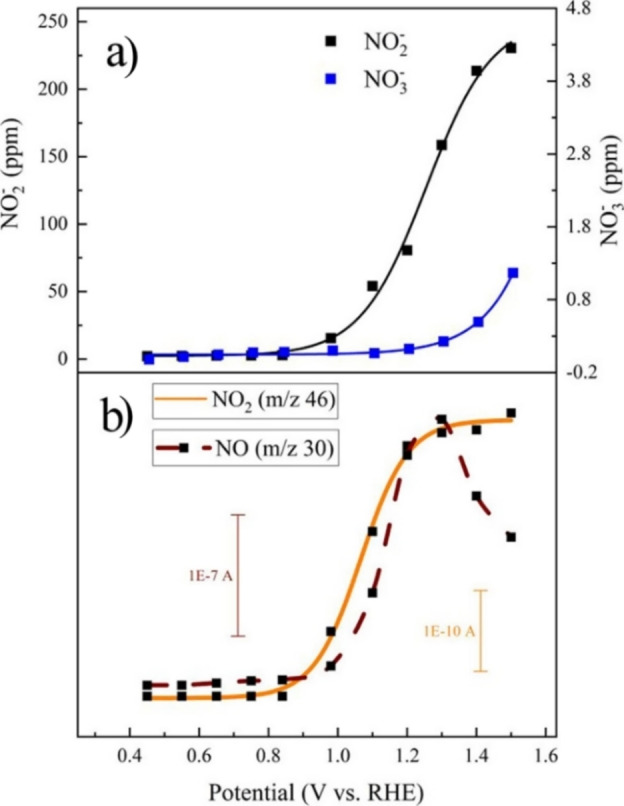
a) Ion chromatography (IC) detection of solution‐phase NO_2_^–^ and NO_3_^–^ species; (b) Mass spectrometry (MS) detection for gaseous NO and NO_2_. Reprinted with permission from Ref. [104], Copyright © 2023, American Chemical Society.

Although Pt‐group metals were commonly investigated for the selective N_2_ formation, they were also shown to form NO_2_^−^/NO_3_^−^. Venturini *et al*. showed that NO_2_^−^ and NO_3_^−^ formation became detectable at 0.85 V and 1.15 V vs. RHE, respectively, with a significant increase in the production at higher oxidative potentials if a polycrystalline Pt catalyst was applied in alkaline media.^[104]^ Mass spectrometry monitoring of potential‐dependent gaseous and solution phase NO_x_ products (Figure 1) demonstrated that NO and NO_2_ react to produce nitrite (3.3). Starting at 1.3 V vs. RHE where NO formation starts to drop (Figure 1b), NO_2_ hydrolysis prompts nitrite and nitrate formation (3.4). Other studies investigating AmOR using differential electrochemical mass spectrometry (DEMS) also discussed the involvement of NO and NO_2_ in nitrite/nitrate formation pathways following equations (3.3) and (3.4).[^100^, ^105^] 
(3.3)

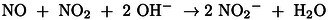



(3.4)

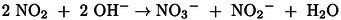




**Figure 2:**
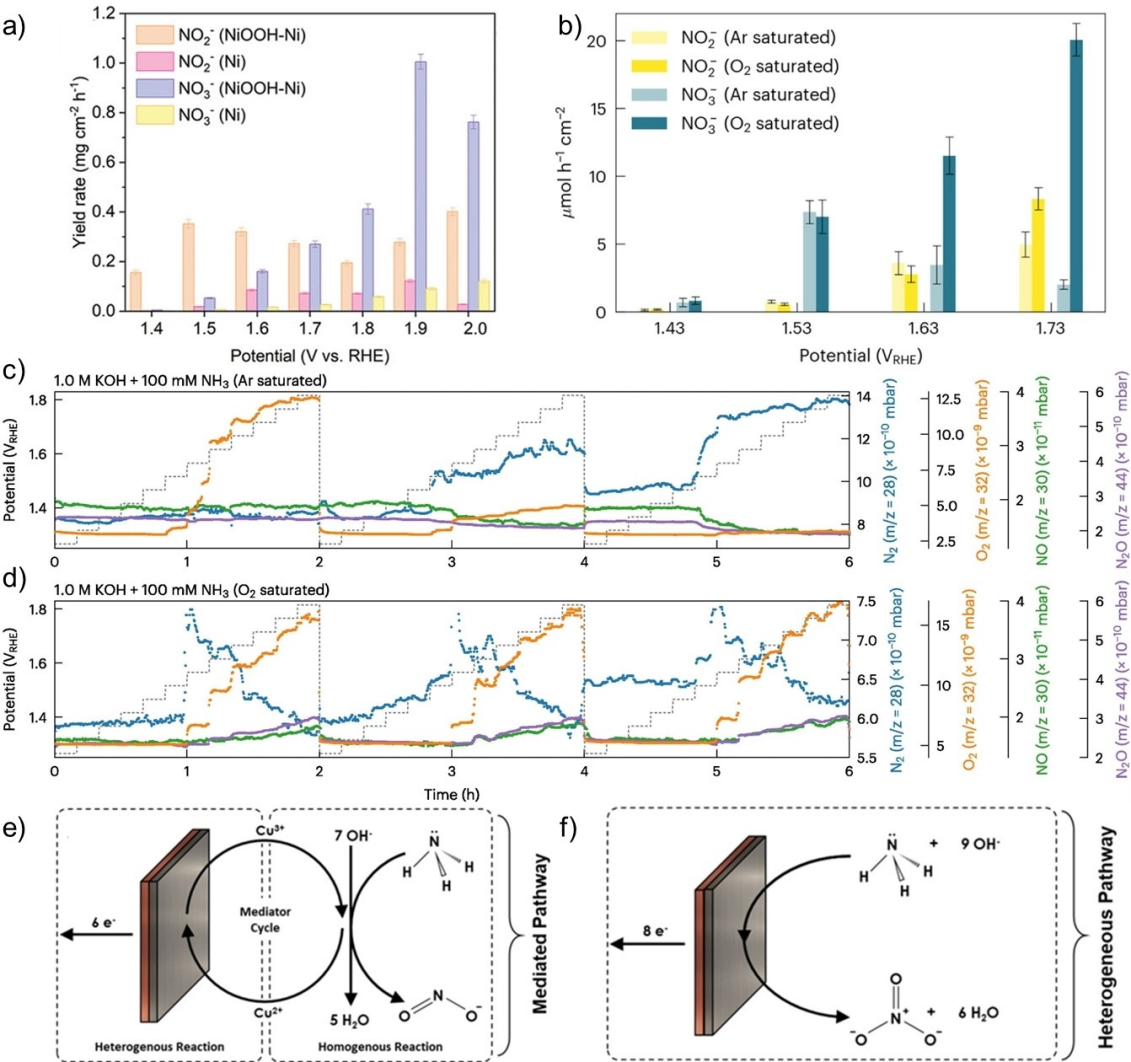
a) Yield of nitrite and nitrate in 0.2 M NH_3_ and 0.1 M KOH as electrolyte using a NiOOH−Ni electrode. Reprinted with permission from Ref. [113], © 2024 The Author(s). Advanced Energy Materials published by Wiley‐VCH. b) NO_2_^−^ and NO_3_^−^ analysis results after 2‐hour AmOR electrolysis conducted in Ar (yellow) and O_2_ (blue) saturated electrolytes (1.0 M KOH + 0.1 M NH₃). DEMS results comparing N_2_, O_2_, NO, and N_2_O in a 1.0 M KOH + 0.1 M NH_3_ electrolyte saturated with c) Ar and d) O_2_. The potential was stepwise increased from 1.27 to 1.82 V vs. RHE in 50 mV steps, each lasting 600 s. Three cycles were conducted. Reprinted with permission from Ref. [117], © 2024 Elsevier Inc. All rights are reserved, including those for text and data mining, AI training, and similar technologies. Proposed AmOR pathways on Cu electrodes: e) mediated and f) heterogeneous mechanism. Adapted with the permission from Ref. [116], © 2021 Wiley‐VCH.

The selectivity and mechanism of AmOR were thoroughly investigated using Ni‐based catalysts. Although Ni(OH)_2_ was discussed as a possible catalytic species participating in AmOR,[^106^, ^107^] the majority of recent studies focused on NiOOH.[^108^, ^109^, ^110^] It was also reported that Ni(OH)_2_ undergoes oxidation from Ni^2+^ to Ni^3+^ to form NiOOH, which acts as an active species participating in the reaction.[^111^, ^112^] A study by Liu *et al*. also indicated that significantly more NO_2_^−^ and NO_3_^−^ can be formed using NiOOH compared to Ni as a catalyst (Figure 2a). The importance of the catalyst structure was highlighted by performing *operando* X‐ray absorption fine structure (XAFS) measurements, which confirmed that Ni^3+^ was the active site during AmOR.^[113]^ Choueiri *et al*. performed a first‐principles simulation study of the AmOR mechanisms on β‐Ni(OH)₂ (0001) which suggested that N₂ formation follows the Gerischer–Mauerer mechanism through NH–NH coupling. In contrast, the formation of NO_x_^−^ involves the deprotonation of NH_3_ to produce adsorbed nitrogen (*N), which then undergoes further reaction with OH^−^.^[114]^ Later the research was extended to a computational study on the β‐NiOOH (0001) surface, and the results were compared with the ones obtained for β‐Ni(OH)_2_ (0001).^[115]^ The NiOOH surface exhibited lower computed overpotentials for N_2_ and NO_2_^−^ formation compared to Ni(OH)_2_. However, due to the stabilization of the nitrate‐forming intermediate (*NO_2_), NiOOH did not show a significantly superior activity compared to Ni(OH)_2_. A density functional theory (DFT) based study by Johnston *et al*. investigated how doping with various metals (Cr, Co, Cu, Fe) in the β‐Ni(OH)_2_ structure affects the limiting potentials for N_2_, NO_2_^−^, and NO_3_^−^ formation.^[118]^ The dopants altered reaction pathway energies, making different reaction intermediates more or less favorable for a specific product formation. Interestingly, at the studied conditions, the limiting potential for nitrite and nitrate formation could not be improved with any of the dopants, whereas Cr and Co were beneficial for N_2_ formation.

As NO_2_^−^ and NO_3_^−^ were commonly reported to be detectable at relatively high oxidative potentials which suggest a substantial contribution to OER, the role of oxygen during AmOR was also discussed. Based on observations using multiple in‐situ characterization techniques Chen *et al*. reported that in Ar saturated 1 M KOH electrolyte AmOR to N_2_ pathway actively competes with OER.^[117]^ Over prolonged operation *NH_x_ species occupying Ni sites caused OER deactivation. This was confirmed using DEMS coupled with cyclic stair electrolysis where a clear deactivation of the O_2_ formation can be observed after the first measurement cycle in Ar saturated electrolyte (Figure 2c). However, in O_2_ saturated electrolyte, the presence of O_2_ mitigated the OER deactivation (Figure 2d) and promoted the formation of NO_X_, NO_2_^−^ and NO_3_^−^ species at high oxidative potentials (E >1.63 V vs. RHE) (Figure 2b). O_2_ formed by OER in Ar saturated electrolyte could play a similar role as an additional O_2_ source used to saturate the electrolyte, however, the concentrations presumably may be too low to have the same positive effect on NO_2_^−^ and NO_3_^−^ formation. Attenuated total reflection infrared spectroscopy (ATR‐IR) also confirmed that an O_2_‐rich environment assisted the formation and regeneration of NiOOH species that participate in AmOR.

AmOR to NO_2_^−^ and NO_3_^−^ mechanisms have been investigated using non‐Ni‐based catalyst compositions as well. MacFarlane *et al*. showed that by adjusting the potential and pH values, AmOR can take place following so‐called mediated or heterogeneous reaction pathways. In a similar manner as Ni^2+^/Ni^3+^, the mediated pathway enables NH_3_ oxidation through the Cu^3+^/Cu^2+^ pair, which undergoes a repeated oxidation/reduction process (Figure 2e). In the mediated pathway, NO_2_^−^ and not NO_3_^−^ is predominantly formed. In contrast, during a heterogenous pathway, NH_3_ is adsorbed and undergoes a surface‐confined oxidation at the electrode, favoring NO_3_^−^ formation (Figure 2f). Interestingly, direct NO_2_^−^ oxidation to NO_3_^−^ was reported to be negligibly slow.

## 4 AmOR Measurement Conditions

### 4.1 Electrolyte pH

The pH value plays a significant role in altering AmOR selectivity. In most cases, AmOR was investigated in the pH range from 7 to 14, with few studies performed in acidic media. Some of the pH‐dependent selectivity patterns are shown in Table 1. To eliminate the influence of other selectivity‐influencing factors (e. g. crystal structure, measurement conditions), only the data obtained by the same study and using the same catalyst material in different electrolytes is compared.

**Table 1:** Influence of electrolyte pH on AmOR selectivity.^[a]^

Catalyst material	Electrolyte	pH	Potential	Current density	FE^[a]^ (O_2_) %	FE^[a]^ (N_2_) %	FE^[a]^ (NO_2_^−^) %	FE^[a]^ (NO_3_^−^) %	Reference
Cu / Cu(OH)_4_^2−^	0.011 M + 0.1 M NH_3_^[b]^	12.04	1.9 V vs. RHE	‐	‐	‐	4 %	22 %	^[116]^
	0.11 M + 0.1 M NH_3_^[b]^	13.04	1.9 V vs. RHE	‐	‐	‐	39 %	24 %	
	1.1 M + 0.1 M NH_3_^[b]^	14.04	1.9 V vs. RHE	‐	‐	‐	18 %	5 %	
Ni(OH)_2_	0.1 M Na_2_SO_4_ + 0.2 M NH_3_^[b]^ + H_2_SO_4_^[c]^	9	1.9 V vs. RHE	‐	0 %	‐	0 %	54 %	^[112]^
	0.1 M KOH + 0.2 M NH_3_^[b]^	13	1.9 V vs. RHE	‐	23 %	‐	40 %	9 %	
	1 M KOH + 0.2 M NH_3_^[b]^	14	1.9 V vs. RHE	‐	32 %	‐	25 %	5 %	
AgO_x_	0.01 M KOH + 0.1 NH_4_Cl	12	≈1.2 V vs. SHE	5 mA⋅cm^−2^	‐	90 %	8 %	<2 %	^[119]^
	0.1 M KOH + 0.1 M NH_4_Cl	13	≈0.9 V vs. SHE	5 mA⋅cm^−2^	‐	44 %	56 %	<2 %	
	1 M KOH + 0.1 M NH_4_Cl	14	≈0.75 V vs. SHE	5 mA⋅cm^−2^	‐	20 %	84 %	<2 %	

^[a]^ Values were extracted based on visual analysis of the available figures in the supplied references. Therefore, small errors (1‐2 %) could be anticipated. ^[b]^ Concentrated ammonia solution was employed for the electrolyte preparation. ^[c]^ Electrolyte adjustment to the predefined pH using sulfuric acid.

In the already mentioned study by MacFarlane and co‐authors,^[116]^ NO_3_^−^ formation is reported to become more prominent over NO_2_^−^ formation at lower pH (0.011 M KOH). However, despite the pH adjustment NO_3_^−^ can only be observed when E vs. RHE >1.7 V. At electrolytes with higher pH (0.11 M and 1.1 M KOH) NO_2_^−^ formation dominated over NO_3_^−^. This can be expected, as it is suggested that NO_2_^−^ is formed through NH_3_ oxidation at a Cu^3+^ tetrahydroxy species and Cu oxidation would be more prominent in a strongly alkaline environment. However, at higher oxidation potentials and at higher electrolyte concentrations (1.1 M KOH) competition with OER increases, concomitantly altering selectivity patterns (Table 1). In an AmOR selectivity study using electrolytes in the pH range 9–14, Medvedev *et al*.^[112]^ concluded that NO_3_^−^ becomes the dominant product at pH <12, with the highest FE of ≈54 % at pH 9–10 and 1.9 V vs. RHE. NO_3_^−^ was already detectable at 1.6 V vs. RHE. NO_2_^−^ became dominant at pH >12, with the highest FE value of ≈ 40 % obtained at pH 13 and at 1.9 V vs. RHE. At pH 14 the selectivity became favorable towards O_2_ formation (FE O_2_ ≈32 %, 1.9 V vs. RHE) (Table 1). Surprisingly, in a recent work by Vu *et al*.,^[119]^ lowering the pH did not yield a favo‐rable NO_3_^−^ selectivity, even though the NO_2_^−^ selectivity increased with increasing pH of the electrolyte (Table 1). Although relatively high potential values (≈1.2 V vs. SHE in 0.01 M KOH) were recorded, it is possible that for the investigated Ag‐based catalyst the applied potential was insufficient to promote NO_3_^−^ formation.

Kapałka *et al*.^[107]^ confirmed that AmOR on a Ni electrode is pH dependent and proceeds mainly at pH >7, where NH_3_ rather than NH_4_^+^ participates in the oxidation reaction. It is important to note that during 12 h electrolysis at 20 mA⋅cm^−2^ at pH 11 only NO_3_^−^ could be identified as aqueous AmOR product, and the NO_2_^−^ concentration remained below the detection limit. These results are in agreement with the ones obtained by Shih *et al*.,^[111]^ where at pH 11 NO_3_^−^ was formed at significantly higher amounts compared to NO_2_^−^ on Ni‐based electrodes. Using various Ni foam/CuCo electrodes for AmOR at pH 11, Tsai *et al*. observed only negligible amounts of NO_2_^−^ and high selectivity towards NO_3_^−^ as well.^[108]^ The reverse trend was obtained by Jiang *et al*. using a NiCu catalyst in 0.1 M KOH. Up to 98 % of NH_3_ was selectively oxidized to NO_2_^−^ without synthesizing relevant amounts of NO_3_^−^.^[110]^ We also reported that using an AlCoCrCuFe catalyst layer‐coated Ni foam‐based electrodes in 1 M KOH, NO_3_^−^ was only formed at very high oxidative potentials and in relatively low amounts (FE NO_3_^−^ = 8 % at 3.8 V vs. RHE).^[120]^ General guidelines can be derived, namely, lower pH electrolytes (pH <12) favor NO_3_^−^ formation, and high alkalinity electrolytes (pH ≥12) are more promising toward NO_2_^−^ production. However, not all examples follow this trend, e. g. Chen *et al*., where on Ni electrodes in 1 M KOH at all investigated potential steps NO_3_^−^ was the dominant aqueous product formed (Figure 2b).^[117]^ These examples demonstrate that AmOR selectivity can be adjusted by dynamic control of the applied electrochemical potential and the electrolyte pH.

### 4.2 Other Stability and Selectivity Altering Factors

Temperature was demonstrated to influence AmOR. Increasing temperature from 25 °C to 55 °C was reported to increase the FE of NO_3_^−^ from 45 % to 77 % using a Ni‐based catalyst. The NO_2_^−^/NO_3_^−^ selectivity can also be altered by using different initial NH_3_ concentrations. It was demonstrated that in the range of 0.03 M – 0.2 M NH_3_ the FE for NO_3_^−^ linearly decreased by around 10 %, whereas the FE for NO_2_^−^ proportionally increased (≈8‐9 %).^[112]^ The corrosive nature of NH_3_ and other reaction intermediates (e. g. NO_2_) should also be considered, as they can lead to surface etching or passivation, which compromises electrode stability.^[121]^ This might especially be problematic in high alkaline electrolytes, as leaching of catalyst material is already a known problem.^[122]^ NH_3_ can readily form amino complexes with transition metals used as catalyst materials for AmOR such as Cu, Co, and Ni, and by this further assists the catalyst dissolution process. Inductively coupled plasma optical emission spectrometry (ICP‐OES) analysis of post‐measurement electrolytes provided first hints on electrode stability and catalyst dissolution rates.^[116]^ Similar techniques like inductively coupled plasma mass spectrometry (ICP‐MS) could also be employed to monitor catalyst dissolution rate.^[123]^ It should also be considered that although catalyst stability is often discussed as a necessity, at the present state of the literature the majority of studies investigating AmOR for NO_2_^−^ and NO_3_^−^ production have not addressed the long‐term stability and anode material deterioration mechanisms so far.

### 4.3 Setup Considerations for Catalyst Evaluation for AmOR

Various electrochemical cell arrangements have been reported for AmOR investigation, including rotating disk electrode (RDE) voltammetry.[^124^, ^125^] Li *et al*. compared Pt/C, Ir/C, and Rh/C catalyst powders, and it was shown that although Pt/C exhibited the highest initial electrocatalytic activity, it suffered from deactivation due to adsorbed poisoning species. RDE provides multiple advantages, as it is a widely accessible method, requires minimal catalyst material, and offers valuable insights into catalyst performance and reaction kinetics. However, RDE is a lab‐scale method limited to specific electrode designs and generally by low currents. Alternative cell designs and strategies enabling catalyst material tests at more practical conditions are essential, especially in the context of NH_3_ as a possible H_2_ carrier compound.

**Figure 3:**
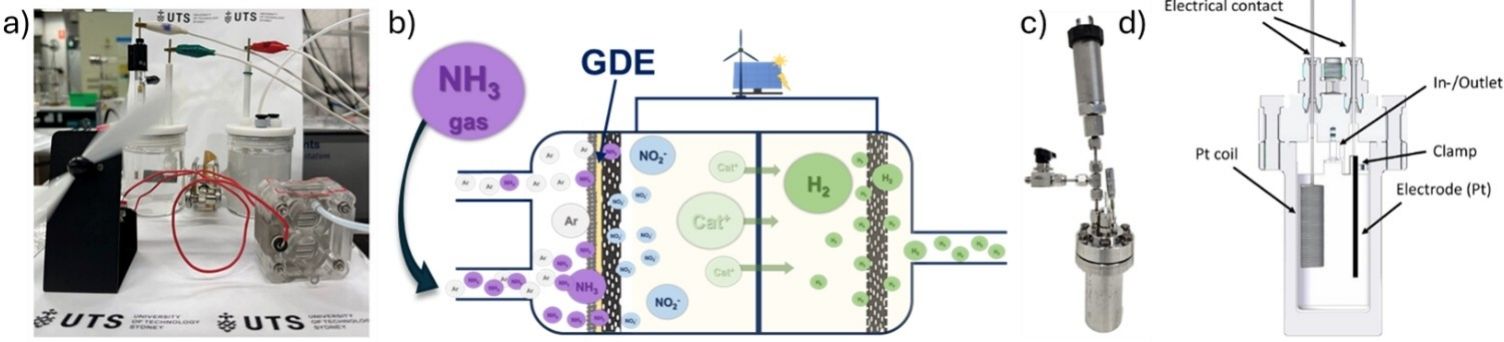
Various experimental setups employed for the oxidation of aqueous, gaseous, and liquid NH_3_. a) Electrolyzer cell used for aqueous NH_3_ oxidation connected to hydrogen fuel cell powering an electric fan. Reprinted with permission from Ref. [113], © 2024 The Authors. Advanced Energy Materials published by Wiley‐VCH GmbH. b) Scheme of gaseous NH_3_ oxidation in an electrolyzer with gas diffusion electrode. Adapted with permission from Ref. [120], © 2024 The Authors. Angewandte Chemie International Edition published by Wiley‐VCH GmbH. c) Picture of the closed electrochemistry autoclave and d) scheme of the high‐pressure, high‐temperature electrochemistry autoclave used for liquid NH_3_ oxidation. Adapted with permission from Ref.^[129]^ Copyright © 2024 The Authors. Published by the American Chemical Society.

Ni foam has been reported as an advantageous material for electrode production for AmOR to NO_2_^−^/NO_3_^−^ due to its high surface area, excellent electrical conductivity, and robust structural support.[^108^, ^126^] Liu *et al*.^[113]^ investigated Ni foam‐based electrodes in a conventional H‐cell, where the counter electrode (HER) and working electrode (AmOR) compartments were separated using an anion exchange membrane. For a proof‐of‐concept, hydrogen generated at the cathode was directly connected to a hydrogen fuel cell, successfully generating electricity and powering a small electric fan (Figure 3a). Nitrogen‐phosphorus‐potassium (N−P‐K)–fertilizers are commonly applied in agriculture. Medvedev *et al*.^[112]^ introduced a promising strategy to directly obtain fertilizers by AmOR. Using Ni foam‐based electrodes a 72 h electrolysis was performed using 0.1 M NH_3_ solution in 0.1 M K_2_HPO_4_ electrolyte. After the measurement they successfully detected NH_4_NO_3_ in 0.1 M K_2_HPO_4_, forming a basis for N−P‐K‐fertilizers. The majority of the studies employed aqueous NH_3_ or aqueous NH_3_ precursors as the source of NH_3_, which would require additional processing of the liquefied NH_3_ used for transportation. The liquefied NH_3_ could be easily converted back to its gaseous form at room temperature and normal pressure. Hence, direct oxidation of NH_3_ gas would be beneficial for the overall simplicity of the process. Based on this, we proposed a proof‐of‐concept electrolyzer model system, where gaseous NH_3_ was directly oxidized on catalyst‐coated Ni foam‐based gas diffusion electrodes (GDEs) (Figure 3b).^[120]^ Naturally, oxidation of gaseous ammonia could also be realized by purging NH_3_ (g) through the electrolyte; however, GDEs provide a three‐phase boundary between solid catalyst, liquid electrolyte, and gaseous reactant, thus enhancing reactant gas accessibility, improving reaction kinetics, and increasing overall efficiency.

Another valuable strategy not yet discussed in the scope of this work is the oxidation of liquid NH_3_. This approach would enable the oxidation of NH_3_ in the same form as used for transportation. Although this research area is not yet widely explored, liquid NH_3_ oxidation to N_2_ and H_2_ on Pt electrodes has been demonstrated.^[127]^ Little *et al*. additionally demonstrated the successful use of Fe‐based anodes for liquid NH_3_ oxidation as an alternative to Pt. The authors proposed that due to the sufficiently high applied potentials, Fe nitride film can be formed on the surface of the electrode, preventing the dissolution of Fe in the highly corrosive environment and enabling successful AmOR to N_2_ and H_2_.^[128]^ However, the successful formation of NO_2_^−^/NO_3_^−^ in liquid NH_3_ was only recently reported by Krebs and Schüth.^[129]^ It could be demonstrated that through the oxygen reduction reaction at the cathode activated reactive oxygen species participated in AmOR enabling the formation of nitrogen oxides, reaching above 40 % FE values for combined NO_2_^−^/NO_3_^−^. This work provides a valuable basis for further exploration of liquid ammonia oxidation research and will hopefully accelerate further progress.

Depending on the employed NH_3_ source (aqueous, gaseous, or liquid) significantly different measurement setups are required (Figure 3). On the one hand, measuring the oxidation of aqueous NH_3_ solutions is advantageous due to its simplicity. On the other hand, NH_3_(g) or NH_3_(l) allow lab‐scale research at more industrially relevant conditions. The analysis of gaseous AmOR products like O_2_, N_2_, and cathodically produced H_2_ was commonly performed using gas chromatography. Quantification of NO_2_^−^ and NO_3_^−^ was done either by UV‐vis spectroscopy or ion chromatography. Various *operando* or in situ techniques such as differential electrochemical mass spectrometry (DEMS), Raman spectroscopy, AT‐FTIR spectroscopy, and X‐ray absorption spectroscopy (XAS) were reported to contribute to the elucidation of the AmOR mechanism.[^66^, ^71^, ^104^, ^110^, ^113^, ^117^, ^119^]

## 5 Electrocatalyst Materials for Nitrite/Nitrate Formation During AmOR

Synthesis methods for the preparation of AmOR electrocatalyst materials have already been summarized previously and are therefore not in‐depth discussed in the present work.[^38^, ^40^] Although substantial progress has been made investigating alkaline AmOR for the formation of valuable products such as NO_2_^−^ and NO_3_^−^, many challenges remain. Highly selective nitrite‐forming catalysts have been reported, with FE close to and above 90 %. However, similarly high selectivity towards nitrate formation has not been achieved yet. An overview of non‐noble metal catalysts for the formation NO_2_^−^/NO_3_^−^ is summarized in Table 2, together with the applied potential and employed electrolyte as selectivity‐influencing factors, and the highest FE values for NO_2_^−^ and NO_3_^−^.

**Table 2:** Summary of non‐noble electrocatalyst materials for NO_2_^−^/NO_3_^−^ formation during AmOR.^[a]^

Catalyst material	Ref.	Maximum selectivity towards NO_2_^−^ formation				Maximum selectivity towards NO_3_^−^ formation			
		FE ^[a]^ (NO_2_^−^) %	Selectivity ^[a]^	Electrolyte	Measurement potential / current	FE ^[a]^ (NO_3_^−^) %	Selectivity ^[a]^	Electrolyte	Measurement potential / current
Ni foam / AlCoCrCuFe	^[120]^	88 %	‐	1 M KOH	1.51 V vs. RHE / 50 mA⋅cm^−2^	8 %	‐	1 M KOH	3.8 V vs. RHE / 200 mA⋅cm^−2^
Ni foam / β‐NiOOH / CoO_x_H_y_	^[126]^	≈38 %	‐	0.1 MK_2_HPO_4_ + 0.5 M NH_3_^[b]^	1 V vs. SHE	≈41 %	‐	0.1 M K_2_HPO_4_ + 0.5 M NH_3_^[b]^	1 V vs. SHE
Ni_0.8_Cu_0.2_	^[110]^	98 %	‐	0.1 M KOH + 0.005 M (NH_4_)_2_SO_4_	1.48 V vs. RHE	‐	‐	‐	‐
C fibre / Ni	^[69]^	8±2 %	‐	0.1 M KOH + 0.1 M NH_3_^[b]^	2±0.003 V vs. RHE	19±7 %	‐	0.1 M KOH + 0.1 M NH_3_^[b]^	2±0.003 V vs. RHE
Cu / Cu(OH)_4_^2−^	^[116]^	≈86 %	‐	0.011 M KOH + 0.1 M NH_3_^[b]^	1.7 V vs. RHE	≈45 %	‐	0.011 M KOH + 0.1 M NH_3_^[b]^	1.8 V vs. RHE
[Cu(bipyalkH)L](OTf)_2_	^[132]^	28.5 %	‐	0.137 M (NH_4_)_2_SO_4_ + NaOH ^[c]^	1.6 V vs. NHE	65.2 %	‐	0.137 M (NH_4_)_2_SO_4_ + NaOH ^[c]^	1.6 V vs. NHE
Ni foam / NiOOH	^[113]^	90.4±1.8 %	‐	0.1 M KOH + 0.2 M NH_3_^[b]^	1.4 V vs. RHE	34.6±1.1 %	‐	0.1 M KOH + 0.2 M NH_3_^[b]^	1.9 V vs. RHE
Ni foam / Ni(OH)_2_ / β‐NiOOH	^[112]^	60 %	‐	0.1 M KOH + 0.2 M NH_3_^[b]^	1.6 V vs. RHE	60 %	‐	0.1 M Na_2_SO_4_ + 0.2 M NH_3_^[b]^	2.2 V vs. RHE
Cu_8_Ni_2_	^[133]^	‐	87 %	0.5 M KOH + 0.055 M NH_4_Cl	1.6 V vs. RHE	‐	‐	‐	‐
Co_0.5_Cu_0.5_	^[108]^	‐	‐	‐	‐	‐	≈80 %	0.01 M Na_2_SO_4_ + 50 mg/L NH_4_Cl	1.1 V vs. Ag/AgCl (3 M KCl)
Ni400 (Ni foam‐based)	^[130]^	‐	‐	‐	‐	‐	≈68 %	0.1 M Na_2_SO_4_ + (NH_4_)_2_SO_4_	2 mA⋅cm^−2^
Ag/GO400	^[131]^		≈20 %	0.1 M Na_2_SO_4_ + (NH_4_)_2_SO_4_	0.4 V vs. Hg/HgO	‐	≈67 %	0.1 M Na_2_SO_4_ + (NH_4_)_2_SO_4_	1.4 V vs. Hg/HgO
AgO_x_	^[119]^	86±4 %	‐	0.1 M KOH + 0.1 M NH_4_Cl	1.1 V vs. SHE	‐	‐	‐	‐

^[a]^ Some of the values were not available and had to be extracted based on visual analysis of the available figures in the supplied references. Therefore, errors (1‐2 %) could be anticipated. ^[b]^ Concentrated ammonia solution was employed for the electrolyte preparation. ^[c]^ Adjusted to pH=9 with 1.0 M NaOH.

When the FE values are not available, the selectivity value is provided. For a long time, AmOR was dominated by the narrative that N_2_ is the most desirable product. Therefore, multiple studies that reported initially promising NO_2_^−^/NO_3_^−^ production rates, aimed to modulate the selectivity towards N_2_ production, as their main goal was AmOR for e. g. wastewater treatment.[^130^, ^131^] As reflected in Table 2, Ni, Co, Cu, and Ag are the most frequently used elements investigated for NH_3_ oxidation to NO_3_^−^/NO_2_^−^. However, so far mostly compositionally relatively simple catalysts have been explored.

A more unusual catalyst material was reported by Liu *et al*.^[132]^ A mononuclear Cu electrocatalyst [Cu(bipyalkH)L](OTf)_2_ (bipyalkH=2‐[(2,2’‐bipyridin)‐6‐yl]propan‐2‐ol, L=methanol or acetonitrile) reached high FE value of 94 % for the sum of NO_2_^−^ and NO_3_^−^ formation with no activity for the OER. Johnston *et al*. reported that Ni in 0.1 M KOH demonstrated the highest yields for NO_2_^−^ and NO_3_^−^ formation out of 19 investigated transition metal particles immobilized on carbon fiber.^[69]^ According to the study, Ag, Co, Cu, Fe and other elements were able to produce NO_2_^−^/NO_3_^−^ as well. As demonstrated in Table 2, combining multiple elements (Cu−Ni, Cu−Co, Ni−Co) has already been suggested as a promising strategy to obtain desirable selectivity patterns. It is expected with a further development of the topic in the future more intricate catalyst compositions will be introduced, discovering unexpected synergistic interactions from multi‐metal electrocatalystsIn our previous work, we investigated the selectivity of six compositionally different quinary catalysts for AmOR. Although high FE for NO_2_^−^ (88 %) could be achieved using AlCoCrCuFe as catalyst, we discovered that the catalytic properties were strongly influenced by the presence of Co and Cu, as bimetallic CoCu catalyst could reach up to 88 % FE for NO_2_^−^ as well, only at a slightly higher potential value. Moreover, it was observed that catalyst compositions without Co and Cu in their structure were significantly more selective towards N_2_ formation in comparison to NO_2_^−^.^[120]^

## 6 Summary and Outlook

Ammonia, as a promising hydrogen carrier compound might play a vital role in the green energy sector development. Ammonia utilization in a fuel cell enables energy generation by splitting NH_3_ to N_2_ and H_2_. The same selectivity pattern is expected during wastewater treatment, as the production of N_2_ ensures environmental compatibility. However, for AmOR in an electrolyzer setup, a different selectivity narrative can also be discussed. Ammonia oxidation to NO_2_^−^ and NO_3_^−^, chemicals of great industrial importance, could provide an alternative to the energy‐intensive and NO_x_ oxides emitting Ostwald process. Moreover, anodic AmOR could be coupled with cathodic H_2_ production, generating more H_2_ compared to the traditional 2 e^−^ water electrolysis process. Although Pt was the most widely investigated NH_3_ oxidation catalyst, due to the scarcity and correspondingly high prices of Pt‐group metals, increasingly more focus is on non‐noble or noble‐metal‐lean catalyst compositions. Therefore, we mainly discussed the progress in employing noble metal‐free catalyst compositions for the NH_3_ oxidation to NO_2_^−^ and NO_3_^−^. Although there is no consensus about the exact reaction mechanism, we briefly provided an overview of computational and experimental studies investigating NO_x_ species formation. Ideally, AmOR as an alternative anode reaction would occur at lower anodic overpotentials compared to O_2_ production. However, experimental results indicate that for the formation of NO_2_^−^/NO_3_^−^ relatively high oxidation potentials are necessary, especially in highly alkaline environments. Even though first hints into the role of oxygen during the formation of NO_x_ species are available, the mechanism is not yet fully clear. Therefore, in‐depth studies further clarifying the relation between OER and AmOR are necessary.

Another not well‐understood step is the low NO_3_^−^ production in highly alkaline electrolytes (pH > 12). This is especially interesting as commonly high amounts of NO_2_^−^ can be produced, however, further oxidation of NO_2_^−^ to NO_3_^−^ is sluggish and requires significantly higher anodic potentials. However, it should be noted that this trend was not unanimously observed, raising the question of what distinctive factor is governing the NO_2_^−^ vs. NO_3_^−^ formation even at relatively similar catalyst materials. NO_3_^−^ could be formed at lower alkalinity or neutral pH electrolytes, which also decreases the competition by the OER. Yet, an exclusively NO_3_^−^ forming catalyst material was not reported. It should also be noted, that ensuring efficient H_2_ generation at the cathode remains a crucial element in determining the feasibility of electrolyzer technologies for AmOR. Therefore, employing low‐concentration electrolytes might not be optimal for the HER. Possibly, bifunctional membranes could be applied in the future if different pH values in the cathode and anode compartments are required. The influence of temperature, catalyst stability, and the source of the NH_3_ supply require different measurement setup designs. It is also important to highlight the importance of investigations at industrially more relevant conditions as a way of bridging the lab‐scale research and the future implementation of the technology.

AmOR using non‐noble metal catalyst materials was so far dominated by Ni, Co, Cu, and Ag. The majority of the investigated catalyst compositions were relatively simple – mono‐ or bimetallic. In the future, further screening of new catalyst compositions is necessary for the improvement of NH_3_ energy conversion reactions. Merging the research fields of AmOR and compositionally‐complex multi‐metal catalyst materials is proposed as a potential strategy to use the combinatorial tailoring possibilities in multi‐metal electrocatalysis for the discovery of highly selective and active materials for AmOR under the formation of NO_2_^−^ and NO_3_^−^.

## Conflict of Interests

The authors declare no conflict of interest.

## Biographical Information

*Ieva Agne Cechanaviciute earned her bachelor′s degree in chemistry from Vilnius University in Lithuania in 2019. While pursuing her master‘s degree at the same university, she completed an internship at the Center for Electrochemical Sciences at Ruhr University Bochum under the supervision of Prof. Schuhmann. After finishing her master‘s studies in 2021, she joined Prof. Schuhmann′s research group as a PhD student. She obtained her doctoral degree in 2024 and is currently continuing her work as a postdoctoral researcher at Ruhr University Bochum*.

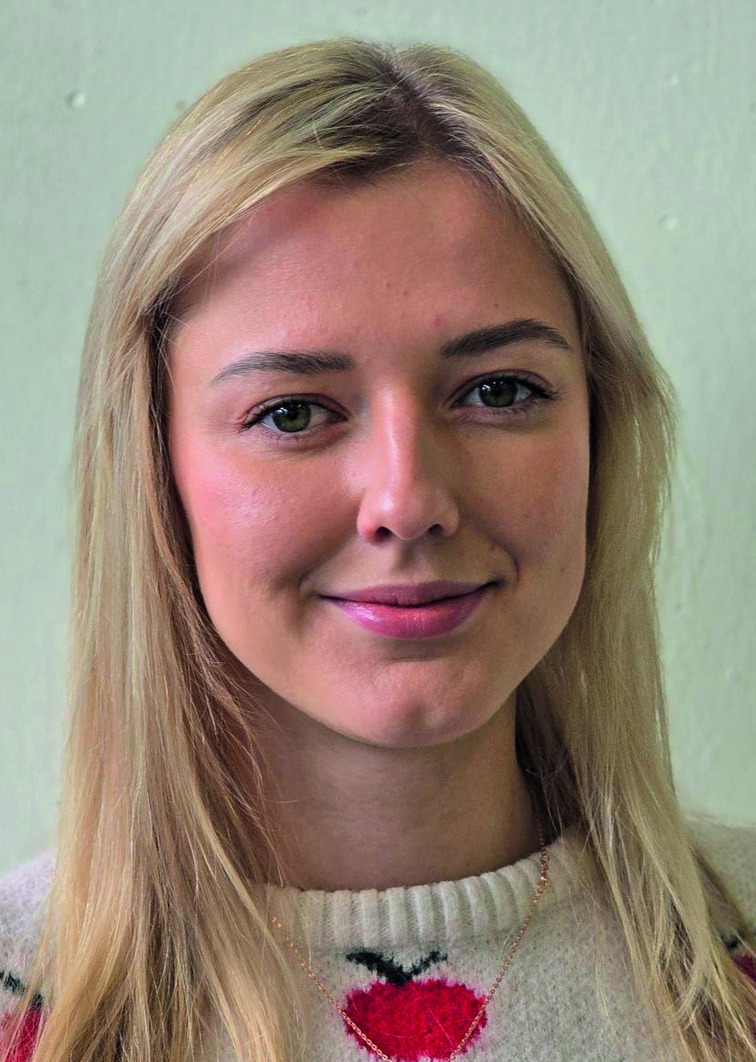

## Biographical Information

*Wolfgang Schuhmann pursued his studies in chemistry at the University of Karlsruhe and earned his Ph.D. in 1986 under the guidance of F. Korte at the Technical University of Munich. After completing his habilitation in 1993 at the same university, he took on the role of professor of Analytical Chemistry at Ruhr University Bochum in 1996. His research encompasses micro‐ and nanoelectrochemistry, scanning electrochemical microscopy and related techniques, biosensors, biofuel cells, and the design and investigation of electrocatalysts for a wide variety of energy conversion reactions*.